# Human papillomavirus-associated anal cancer, vulvar verrucous cancer, and cervical cancer in a post-renal transplant patient-a case report

**DOI:** 10.3389/fonc.2025.1579795

**Published:** 2025-07-04

**Authors:** Xin Feng, Qing-bo Shen, Ke Yuan, Yu Mao, Xia Wang, Ai-ping Min

**Affiliations:** ^1^ Department of Obstetrics and Gynecology, People’s Hospital of Leshan, Leshan, China; ^2^ Department of Proctology, People’s Hospital of Leshan, Leshan, China; ^3^ Department of Pathology, People’s Hospital of Leshan, Leshan, China; ^4^ Department of Traditional Chinese Medicine, People’s Hospital of Leshan, Leshan, China

**Keywords:** human papillomavirus, kidney transplantation, vulvar verrucous cancer, anal cancer, cervical cancer

## Abstract

Human papillomavirus (HPV) can cause tumors at specific anatomical sites in human body. Herein, we report the case of a 73-year-old female patient with a rare presentation of three distinct HPV-associated squamous cell carcinomas (anal, vulvar, and cervical), who had undergone kidney transplantation due to renal failure and was administered long-term cyclosporine, mycophenolate mofetil, and prednisone acetate for 20 years. Evidence of HPV16/59 infection was detected in Evidence of HPV16/59 infection was detected in formalin-fixed paraffin-embedded (FFPE) tissue samples obtained from the patient’s vulvar tumor and anal tumor via PCR-based assays, as well as from exfoliated cells of the cervix. This case confirms the potential of HPV to drive carcinogenesis across multiple ano-genital sites within the same patient. Immunosuppression in transplant patients significantly increases the risk of human papillomavirus (HPV)- associated malignancies; however, the synchronous or metachronous development of triple anogenital cancers in a single individual is exceptionally uncommon. This case highlights the aggressive potential of HPV oncogenesis in immunocompromised hosts and underscores the need for rigorous cancer surveillance in this population.

## 1 Introduction

Human papillomavirus (HPV) is one of the most common sexually transmitted infections. Persistent HPV infection can cause tumors at specific anatomical sites in the human body (1). According to published statistics, over 99% of cervical cancer cases are caused by HPV infection (2). Additionally, HPV can causes cancers of the external genitalia, vagina, anus, and oropharynx (3–7). The HPV virus can integrate into the host cell genome, induce mutations and alter gene expression patterns via E6/E7 gene expression, thereby affecting the integrity and expression of proteins involved in the progression of cancer (8). Integration of the HPV viral genome depends on the type of HPV and infected epithelium; of all sub-types, HPV type 16 is most likely to integrate into the host’s DNA (9). Herein, we report the case of an elderly female, post-renal transplant patient on long-term immunosuppressants, who was diagnosed with vulvar verrucous carcinoma, cervical cancer, and anal cancer. modern diagnostic and therapeutic procedures revealed evidence of HPV infection in all three lesions. The patient’s immunosuppressed state led to a lack of effective antiviral immune response (10), therefore increased the risk of persistent HPV infection and the incidence of HPV-associated cancers (11, 12). This may be the first case report of tumors in multiple regions within the same individual caused by HPV. This case highlights the aggressive potential of HPV oncogenesis in immunocompromised hosts and underscores the need for rigorous HPV infection and cancer surveillance in this population.

## 2 Case presentation

### 2.1 Relevant past interventions

In 2012, a 73-year-old female patient discovered a pea-sized vulvar tumor, that partially protruded from the skin, but was not associated with any symptoms such as redness, swelling, pain, itching, or ulceration. Laser therapy and cryo-therapy were performed on the tumor in 2012 and 2015 respectively. In 2016, the tumor recurred at the primary site, with a similar size and appearance as the initial presentation, but was not treated. In July 2021, the tumor had gradually proliferated, protruded outwards, and exhibited a faster growth rate. The patient purchased and used self-prescribed topical medication; however, the exact name of the medication could not be ascertained. There was no notable improvement in her symptoms, and the tumor surface gradually became ulcerated with minor bleeding; therefore, the patient visited our hospital on January 4, 2022.

### 2.2 Physical examination

Physical examination detected an irregular-shaped tumor on the left side of the external genitalia. The tumor measured 5 * 4cm in its widest diameter, and protruded considerably from the skin. Some parts of the tumor were covered by brown keratinous crusts, while other parts were covered by yellow or white keratinous tissues, with an incomplete epidermis. The color of the surrounding skin was normal, and there was no obvious lymphadenopathy in the groin.

During physical examination, we found a tumor in the anal region, that was not continuous with the vulvar tumor. The anal tumor was around the anus orifice and was brown in color, with cauliflower like changes, unclear boundaries, and surface erosion, accompanied by circular protrusion of hemorrhoids, and subcutaneous varicose veins. The anal tumor was hard and brittle, with poor mobility.

### 2.3 Treatment

**Figure 1:**
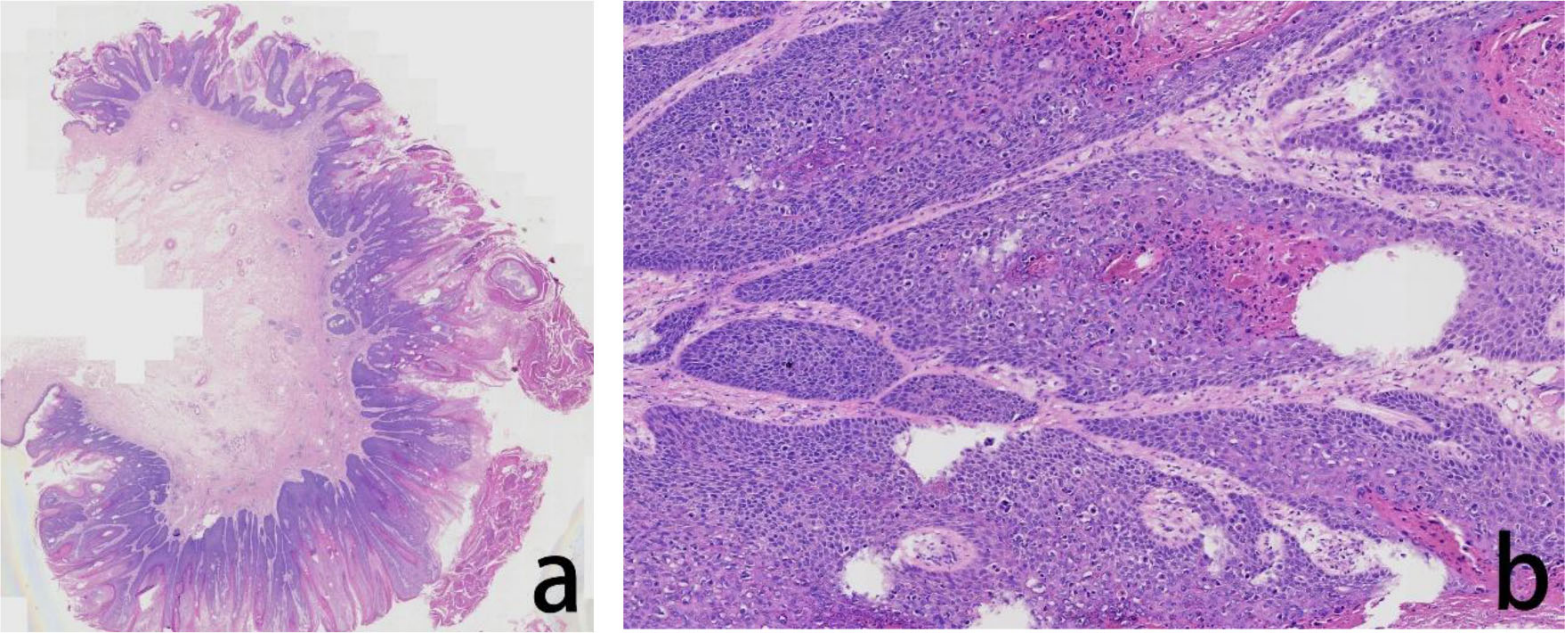
Assessment of the H&E stained vulvar tumor FFPE tissue. The histopathological evaluation in lower magnification, seen in the left panel, revealed exophytic, verrucous growth pattern with prominent hyperkeratosis, parakeratosis, and a ‘pushing’ border **(a)**. In higher magnification, in the right panel, the tumor cells showed atypia, fragmented nuclei, and abnormal keratinization, hollowed out cells were visible **(b)**.

**Figure 2:**
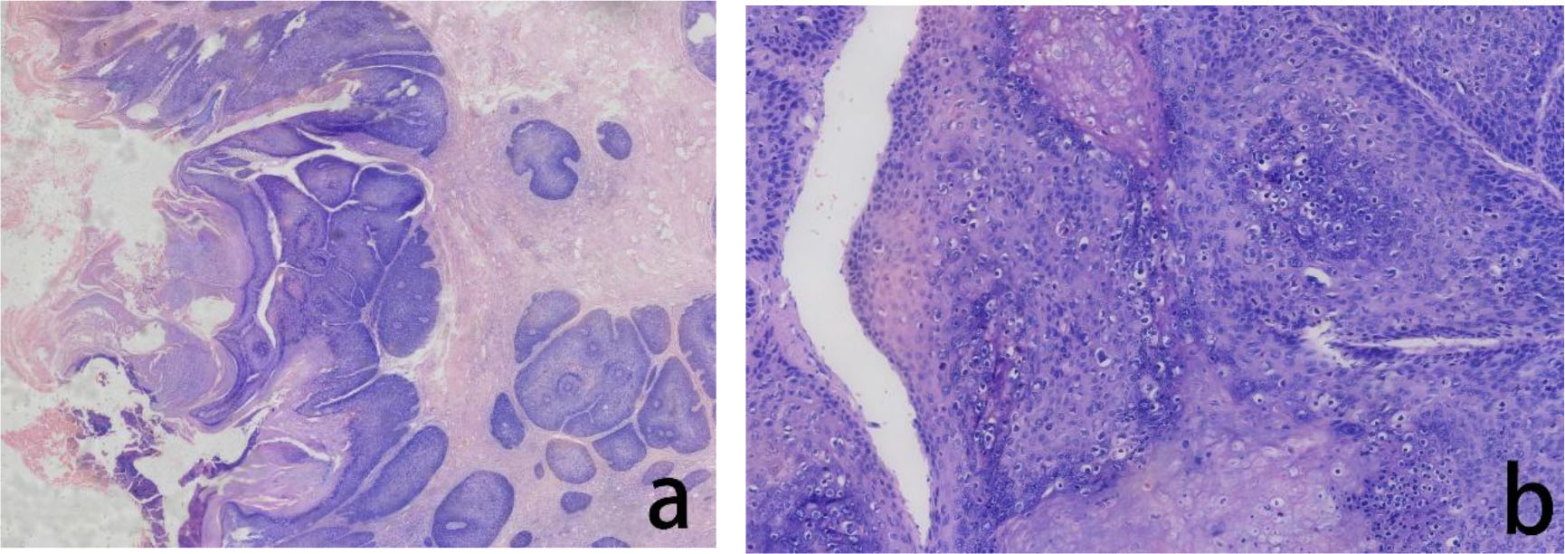
Assessment of the H&E stained anal tumor FFPE tissue. The histopathological evaluation in lower magnification, seen in the left panel, revealed severe squamous epithelial hyperplasia and malignant transformation, accompanied by incomplete keratinization and hyperkeratosis **(a)**. In higher magnification, in the right panel, the tumor cells showed cellular atypia with increased nucleocytoplasmic ratio and visible hollowed out cells **(b)**.

On January 7, 2022, the patient underwent a wide local excision of the skin tumor on the vulva under general anesthesia. Postoperative pathological examination revealed verrucous carcinoma of the vulvar tumor (**Figure 1**). On January 10, 2022, a biopsy of the anal tumor was also performed, and histopathological evaluation revealed severe squamous epithelial hyperplasia and malignant transformation, accompanied by incomplete keratinization and hyperkeratosis. Therefore, on January 20, 2022, the anal tumor was successfully resected. Postoperative pathological examination suggested highly differentiated squamous cell carcinoma (**Figure 2**). On February 21, 2022, postoperative anal cancer radiotherapy was performed, followed by field reduction treatment. Due to a history of kidney transplantation and multiple underlying diseases, the patient and her family decided to abandon chemotherapy and undergo regular follow-up.

### 2.4 Follow-up and new lesions

**Figure 3:**
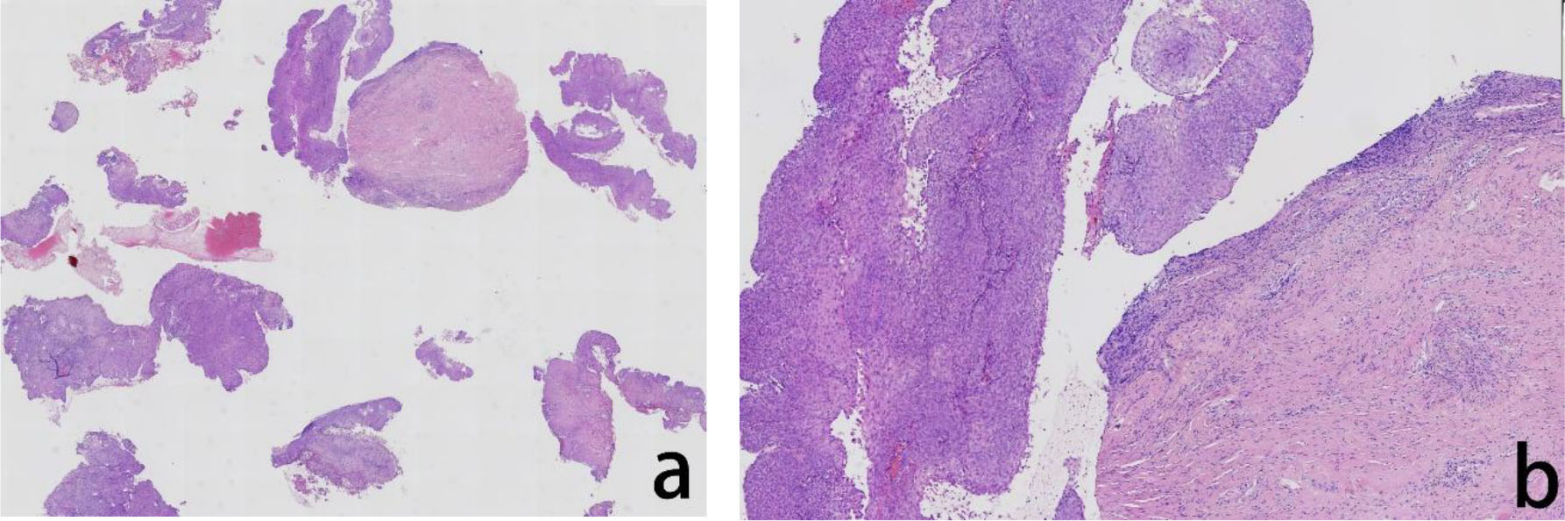
Assessment of the H&E stained cervical biopsied tissue The histopathological evaluation in lower magnification, seen in the left panel, revealed exophytic papillary growth and visible hollowed out cells, indicated high-grade squamous intraepithelial lesion (HSIL) **(a)** In higher magnification, in the right panel, tumor cells showed nuclear division and hollowed out cells were visible **(b)**.

**Figure 4:**
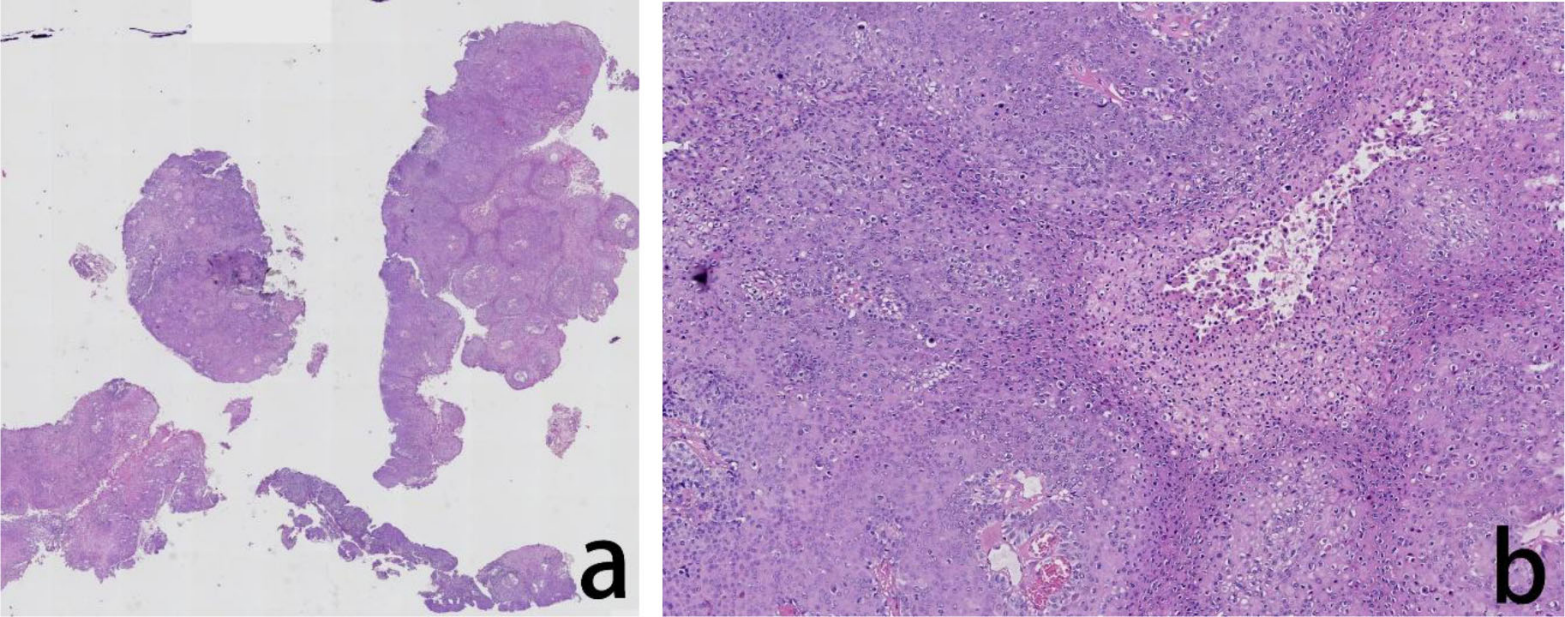
Assessment of the H&E stained vaginal biopsied tissue. The histopathological evaluation in lower magnification, seen in the left panel, revealed exophytic papillary growth and visible hollowed out cells, indicated cervical intraepithelial neoplasia (CIN) III **(a)** In higher magnification, in the right panel, tumor cells showed nuclear division and hollowed out cells were visible **(b)**.

Patient was followed up regularly, no tumors recurrence were detected, and no complications such as anal stenosis occurred at follow-up 2 years postoperatively. On September 20, 2024, the patient visited the gynecology department with a complain of watery vaginal discharge. Physical examination revealed congestion of the vaginal wall and cervix, and, significant contact bleeding from the vaginal wall, PCR-based assays of exfoliated cells of the cervix indicated HPV16/59 (+), Furthermore, liquid-based cytology indicated atypical squamous cells of undetermined significance (ASC-US). At our suggestion, the patients underwent cervical biopsy under colposcopy, which indicated high-grade squamous intraepithelial lesion (HSIL)/cervical intraepithelial neoplasia (CIN) III, but more severe lesions could not be ruled out (**Figure 3**). Biopsy of the vaginal wall indicated a vaginal intraepithelial neoplasia (VaIN) III (**Figure 4**). We explained two treatment options for the patient: Cervical Conization and Total Hysterectomy, while the patient requested treatment at a higher-level hospital and was appropriately referred. On December 3, 2024, the patient underwent a total hysterectomy at another hospital. Postoperative pathological examination revealed widespread cervical CIN III involving glandular tissue, localized microinvasive squamous cell carcinoma in the interstitium, and vaginal wall VaIN II-III. The patient is still on regular follow-up.

### 2.5 Medical history

In 2004, the patient underwent renal transplantation for renal failure at another hospital and was subsequently placed on long-term immunosuppressive therapy, including oral cyclosporine mycophenolate mofetil, and prednisone acetate. In 2014, the patient was diagnosed with hepatitis B virus (HBV) infection (10 years ago) and was prescribed long-term entecavir therapy. In April 2021, the patient developed herpes zoster on her the right lower extremity. In May 2021, she was diagnosed with immunocompromised host-associated pneumonia.

### 2.6 Further examination for HPV

PCR-based assays identified types 16 and 59 HPV in the patient’s exfoliated cervical cells. The FFPE tissue samples prepared from postoperative tumor tissue obtained from the patient’s vulva and anus were sent to a higher-level hospital where*in situ* hybridization (RNA scope) testing was performed. Under light microscopy, positive spot and patchy signals were detected in tumor cells from the vulva and anus. Analysis of both sites confirmed the presence of transcriptionally active HPV at both locations. In Additionally, we conducted HPV typing tests (including 14 high-risk HPV types and 9 low-risk HPV types) on (FFPE) tissue samples prepared from two locations at a higher-level hospital, using polymerase chain reaction reverse dot hybridization as the detection method. The analysis revealed that the vulvar tissue was positive for HPV types 16 and 59, while the anal tissue was positive for HPV types 16,18, and 59. Case timeline and key data were described in detail in **Table 1**.

**Table 1:** Case timeline and key data.

Time	Event	Location	Symptoms/signs	Treatment/Intervention	HPV-Related Testing
2004	Renal transplantation	Kidney	Renal failure, with subsequent long-term immunosuppressive therapy	Renal transplantation	Not documented
2012	Vulvar tumor discovery	Vulva	Pea-sized tumor, no symptoms (redness, pain, ulceration)	Laser therapy	Not tested
2015	Tumor recurrence	Vulva	Similar characteristics	Cryotherapy	Not tested
2016	Tumor recurrence	Vulva	Similar characteristics; untreated	None	Not tested
Jul-21	Rapid tumor growth with ulceration	Vulva	Ulceration and bleeding	Topical self-medication (unknown, ineffective)	Not tested
4-Jan-22	Hospital visit	Vulva,Anus	Vulvar tumor (5cm×4cm), anal cauliflower-like mass	Preoperative evaluation	Not tested
7-Jan-22	Vulvar tumor resection	Vulva	Postoperative pathology: Verrucous carcinoma	Wide excision under general anesthesia	Not tested
10-Jan-22	Anal tumor biopsy	Anus	Postoperative pathology: Severe squamous hyperplasia with malignant transformation	Biopsy	Not tested
20-Jan-22	Anal tumor resection	Anus	Postoperative pathology: Well-differentiated squamous cell carcinoma	Surgical resection	Not tested
21-Feb-22	Postoperative radiotherapy	Anus	Radiotherapy with field reduction	Radiotherapy	Not tested
20-Sep-24	Colposcopy and biopsy	Cervix, Vagina	watery vaginal discharge, Pathological results:HSIL/CIN III (cervix), VaIN III (vagina)	Colposcopy and biopsy	Cervical:HPV16/59(+)
3-Dec-24	Total hysterectomy	Cervix, Vagina	Cervical CIN III with microinvasive squamous cell carcinoma; VaIN II-III	Total hysterectomy	Vulva: HPV16/59, Anus: HPV16/18/59(+)

## 3 Discussion

Vulvar verrucous carcinoma is a unique, highly differentiated, low-grade malignant squamous cell carcinoma, that is generally considered as a rare subtype of squamous cell carcinoma unrelated to HPV infection (13, 14). However, some studies suggest that this condition may be related to type 6, 11, 16, and 18 of HPV. Evidence for HPV infection has been detected in some cases of vulvar verrucous carcinoma, especially in immunocompromised populations. For example, Rando R F et al. detected type 6 HPV in tissue from a patient with vulvar verrucous carcinoma (15), Cuesta K H et al. tested six cases of HIV- positive vulvar verrucous carcinoma; five patients were positive for HPV types 6, 11, 16, and 18 in the cancerous tissue (16). In our present case, we detected HPV types 16 and 59 in the vulvar tissue; these findings were consistent with previous literature. Our patient also had anal cancer; almost all cases of anal squamous cell carcinoma are caused by HPV and progresses from highly squamous intraepithelial lesions. The International Society of Anal Oncology has recommended high-risk HPV for the screening anal cancer (17). The risk factors for anal cancer include HPV infection, a history of anal or sexual intercourse; a history of cervical, vulvar, or vaginal cancer; immune suppression following solid organ transplantation or HIV infection; hematological malignancies; certain autoimmune diseases, and smoking (18). Our patient had multiple risk factors, including HPV infection, vulvar cancer, and a history of solid organ transplantation.

High-risk HPV testing has been validated alone as a primary screening method for cervical cancer detection. The US Health and Human Services recommends that for immunocompromised populations, the strategy for cervical cancer screening should comply with the screening strategy for patients with the human immunodeficiency virus (HIV) in that the interval between cervical cancer screenings should be reduced (19, 20), and vulvar inspection at the time of cervical cancer screening is also very necessary (21). The International Society for Anal Oncology (ISAO) endorses high-risk HPV testing as a standalone screening modality for anal cancer. For organ transplant recipients, it is recommended to commence screening for anal cancer 10 years after transplantation (17). A study from Australia showed that large reductions in cervical cancer incidence have been observed following the cytological and HPV testing programs (22). A cross-sectional study in kidney transplant recipients indicated that High-risk HPV and HSIL testing may identify kidney transplant recipients at higher risk of anal cancer (23). Unfortunately, our patient did not receive regular HPV testing from an early timepoint; thus, we are unable to determine when the patient was first infected with the HPV virus. If regular cervical and anal HPV testing can be implemented even earlier after kidney transplantation following the detection of anal and vulvar verrucous cancer, with regular review and follow-up, this practice may significantly reduce the probability of the occurrence of anal, cervical and vulvar cancers.

Currently, the HPV vaccine has been shown to be very effective in clinical trials and has been widely promoted and applied in China. A 10-year study conducted in Sweden and involving 1.7 million women aged between 10 and to 30 years, revealed that when compared to women who did not receive the HPV vaccine, vaccination before the age of 17 years reduced the incidence of cervical cancer by 88%. However, vaccination between the ages of 17 and 30 years reduced the incidence of cervical cancer by approximately 50% (24). Okunade K S reported that the HPV vaccine may reduce the incidence of vulvar cancer by approximately 50% and intraepithelial lesions of the lower genital tract by at least two-thirds (25). In view of the high HPV infection rate, and the incidence of anal and genital cancer in kidney transplant recipients, Chin-Hong, Peter V suggested that vaccination should be carried out before kidney transplantation in recipients of suitable age (26).

Immunosuppression is a high-risk factor for HPV infection and persistent infection, considerably increasing the incidence of anal, genital, and oral cancers in immunocompromised patients (27). A previous meta-analysis of patients undergoing large-scale organ transplantation revealed that the standardized incidence rate of HPV-related anal and genital cancers was elevated in these patients (28). It has been suggested that one of the mechanisms by which HPV causes cervical lesions in immunosuppressed patients is the immune system’s inability to eradicate the virus, leading to its persistence. If this hypothesis is correct, immunosuppressed individuals exposed to carcinogenic viruses would have an increased risk of persistent HPV infection and tumors development (29). A meta-analysis shows that Immunosuppression permits uncontrolled replication of oncogenic HPV subtypes (e.g., HPV16/18), which disrupt tumor suppressor pathways (p53/Rb) via E6/E7 proteins. Coinfection with multiple HPV subtypes may accelerate multifocal lesions (30). Our patient had a history of kidney transplantation, had been taking immunosuppressants and glucocorticoids for an extended period, and also had hepatitis B, requiring long-term antiviral treatment. Furthermore, as an elderly female with immunosuppression, she was at high- risk of persistent HPV infection and tumor development.

The findings of this study derived from a single patient case, which inherently restricts the ability to extrapolate conclusions to broader populations. The unique interplay of host factors, immunosuppressive regimens, and HPV-related oncogenesis in this case may not fully capture the heterogeneity observed in other post-transplant patients. While the clinical management and short-term outcomes are described, the absence of extended follow-up limits insights into long-term recurrence risks, immune reconstitution effects, or secondary malignancies. This gap hinders a comprehensive understanding of the dynamic relationship between sustained immunosuppression and HPV-driven carcinogenesis.

## 4 Conclusion

In summary, our experience with this case indicates that HPV can cause multiple HPV-related tumors in the anogenital region in the single individual, in immunocompromised populations. Thus, in this population, when diagnosing an HPV-related tumor, it is imperative to remain vigilant for other potential HPV- related tumors. HPV testing and follow-up should begin as early as possible, with timely intervention when lesions are detected. Additionally, promoting HPV vaccines is of great importance. These strategies may have important implications for reducing the incidence of HPV related tumors in such populations.

## Data Availability

The original contributions presented in the study are included in the article/supplementary material. Further inquiries can be directed to the corresponding author.
